# Preventive Effect of Garlic (*Allium sativum L*.) on Serum Biochemical Factors and Histopathology of Pancreas and Liver in Streptozotocin- Induced Diabetic Rats

**Published:** 2013

**Authors:** Fatemeh Masjedi, Ali Gol, Shahriar Dabiri

**Affiliations:** a *Student Research Committee and Department of Physiology, Shiraz University of Medical Sciences, Shiraz, Iran.*; b *Department of Biology, Faculty of Sciences, Shahid Bahonar University of Kerman, Kerman, Iran.*; c *Department of Pathology, Afzalipour Medical School, Kerman, Iran. *

**Keywords:** Diabetes, Garlic, Hepatic enzymes, Histopathology, Serum lipids

## Abstract

Antidiabetic action of garlic is established in animal studies. Since all of the pervious studies have focused on the therapeutic role of garlic, this study investigated the preventive effect of garlic juice on biochemical factors and histological features in Streptozotocin (STZ)- induced diabetic rats.

Forty male rats were divided into five groups (n = 8): 1-Normal group (N), 2-Normal+Garlic group (N+G) received garlic juice (1 mL/100g BW) for 6 weeks, 3-Diabetic group (D) was injected with STZ (60 mg/kg, IP), 4-Diabetic+Garlic-before group (D+G_b_) received garlic juice for 3 weeks before STZ injection and continued for another 3 weeks, 5-Diabetic+Garlic-after group (D+G_a_), three days after STZ injection, they received garlic juice for 3 weeks. Serum biochemical factors were measured by the enzymatic methods and H&E stained sections of pancreas and liver were prepared for light microscopy.

In diabetic rats, elevated levels of glucose, cholesterol and triglycerides, the increment of the activities of ALT and AST, increased food and water consumption were observed. The abnormal increases were significantly (p *< *0.05) decreased in D+G_b_ groups compared to D group. In D group, scattered degeneration of the hepatocytes with lymphocytic infiltration in the portal areas, decrease of pancreatic islets numbers and diameter, atrophy of pancreatic islets were observed. These abnormal histological signs were dramatically ameliorated in D+G_b_ group compared to D group. In D+G_a_ group compared to D+G_b_ group slighter effects of garlic juice on histopathological and biochemical changes were seen.

These results indicate that garlic juice may help in the prevention of the complications of diabetes.

## Introduction

Diabetes is a metabolic disturbance that gradually affects the function of various systems in the body. Poorly controlled blood glucose is believed to be the most important factor in the development of diabetic complications in both type 1 and type 2 diabetes (1). Individuals affected by diabetes are prone to the long-term complications such as retinopathy, cataract, neuropathy, atherosclerosis, nephropathy, embryopathy, and delayed healing of wounds (2).

A worldwide survey reported that diabetes affects nearly 10% of the world population (3). According to a projection of the International Diabetes Federation, 194 million people had diabetes in 2003, which will increase to 333 million by 2025 (4). It is likely to remain a significant threat to public health in the years to come. In the absence of effective and affordable interventions for either type of diabetes, the frequency of the disease will escalate worldwide, with a major impact on the populations of developing countries (5). In modern medicine, the beneficial effects of standard medications on glycemic levels are well documented; the preventive activity of medications against the progressive nature of diabetes and its complications was modest and not always effective. Insulin therapy affords effective glycemic control, yet its shortcomings, such as ineffectiveness on oral administration, short shelf-life; the requirement of constant refrigeration, and in the event of excess dosage, fatal hypoglycemia limits its usage (6).

In spite of the fact that insulin has become one of the most important therapeutic agents known to medicine, researchers have been making efforts to find insulin substitutes from synthetic or plant sources for the treatment of diabetes (7). Herbs and spices have a traditional history of use, with strong roles in cultural heritage, and in the appreciation of food and its links to health (8). 

Medicinal plants continue to provide valuable therapeutic agents, in both modern medicine and in traditional system. The doubts about the efficacy and safety of the oral hypoglycemic agents have promoted a search for safer and more effective drugs for the treatment of diabetes (9). 

Garlic (*Allium sativum *L.) -a common food spice, belongs to the Alliaceae family, which is consumed all over the world as a food flavoring agent and in traditional medicine to enhance physical and mental health (10). 

Garlic and its constituents prepared by various means have been shown to have diverse biological activities, including anticarcionogenic, antiatherosclerotic, antithrombotic, antidiabetic, and various other biological actions (11, 12).

In the 1970s, Jain *et al*. (13) and Jain and Vyas, (14) showed that the ingestion of garlic juice resulted in better utilization of glucose in glucose tolerance test performed in rabbits. The ethyl alcohol, petroleum ether and ethyl ether extracts of garlic produced a significant fall in blood sugar levels in rabbits. Augusti and Sheela, (15) and Sheela and Augusti, (16) consistently showed that S-allyl cysteine sulfuxide (alliin), a sulfur containing amino acid in garlic has a potential to reduce diabetic condition in rat almost to the same extent as did glibenclamide and insulin.

Thus, the antidiabetic action of garlic established in animal studies provided a background for further investigations concerning possible clinical implications for garlic-based preparations. Since all of the pervious studies have focused on the therapeutic role of garlic and garlic preparations in diabetes mellitus, we investigated the preventive effect of oral administration of garlic juice on the changes of biochemical factors and histopathological alterations in pancreatic and hepatic tissue caused by diabetes.

## Experimental

*Animals and groups*

Six-week-old weanling male rats (Wistar strain) weighing 250±20 g were purchased from the Animal House Unit of the Faculty of Sciences, Shahid Bahonar University of Kerman, Kerman, IR Iran. The animals were kept under a 12 h light-dark cycle at an ambient temperature of 23-25 °C and were housed in standard metallic cages where food and water were provided *ad libitum*. Rats were fed pellets consisting of 21% crude protein, 5.5% crude fiber, 6.5-7% crude fat, 0.5% NaCl, 0.7% mixture of minerals (Manganese, Zinc, Iron, Copper, Cobalt, and Selenium), and vitamins (B2, B1, K, E, D3, A), and 2750 kcal./Kg diet [Rodent Diet; Javaneh, Khorasan].

All animals were allowed to adapt to the environment for 1 week after their arrival before the experiment started. The rats received humane care according to the criteria outlined in the “*Guide for the Care and Use of Laboratory Animals*” prepared by the National Academy of Science and published by The National Institutes of Health (17).

In this experiment, forty male rats were divided into five groups of 8 rats each. 1) Normal group (N) received distilled water for 6 weeks and served as a negative control, 2) Normal + Garlic group (N+G) received 1 mL of garlic juice/100 g BW/day (equivalent to 0.4 g/100 g BW) by gastric gavage using ball-tipped needle for 6 weeks, 3) Diabetic untreated group (D) was injected with a single dose of streptozotocin (STZ) (60 mg/Kg BW, IP) and received distilled water for 6 weeks and served as a positive control, 4) Diabetic + Garlic-before group (D+G_b_) received garlic juice for 3 weeks before STZ injection and continued for another 3 weeks and served as preventive group, 5) Diabetic + Garlic-after group (D+G_a_) received garlic juice for 3 weeks and was after injected with STZ and served as therapeutic group. The experimental period for each rat was 6 weeks. Body weight of animals by balance (accuracy: 0.01 g) per day were measured and at the end of the experiment, all animals were housed for 24 h in metabolic cages while they were given free access to water and a standard powdered diet [Rodent Diet; Javaneh, Khorasan]. Their food & water consumption also were carefully estimated. 

*Preparation of garlic juice*

Fresh garlic (*Allium sativum *Linn) bulbs were purchased from the local market in Kerman, Iran, peeled, washed, and chopped into small pieces. 

The garlic juice was prepared by adding 100 g of garlic with 250 ml of distilled water and crushed in a mixing machine. The resultant slurry was squeezed and filtered through a fine cloth and the filtrate was quickly frozen at -10 °C until used (18).

*Induction of experimental diabetes*

Streptozotocin was purchased from Sigma (St. Louis MO, USA). Diabetes was induced with a single dose of STZ (60 mg/Kg body weight, IP); dissolved in 0.9% saline, immediately before use (19). After 3 days of STZ treatment, serum glucose levels were determined in blood samples collected from the retro-orbital plexus of rats after being lightly anesthetized with ether (20). Animals showing fasting blood glucose level >300 mg/dL were considered diabetic (21).

*Biochemical analysis of blood samples*

At the end of the experimental period, rats were fasted for 12 h with free access to drinking water, and then sacrificed by decapitation and blood samples were collected from the sacrificed animals quickly and carefully and put into sterile plastic tubes and allowed to clot at the room temperature for 20 min, then centrifuged at 860 g for 15 min and platelet-free unhemolyzed serum was collected and stored at -30 °C till measurements. Also, pancreas and liver were immediately removed and placed in labeled beakers in 10% formaldehyde solution.

Stored serum samples were analyzed for glucose levels by enzymatic colorimetric method [glucose oxidase-peroxidase method (GOD)]. Cholesterol was measured by enzymatic colorimetric method [cholesterol oxidase-peroxidase method (CHOD)]. Triglycerides (TG) were determined by enzymatic hydrolysis of triglycerides with subsequent determination of liberated glycerol by colorimetry. Serum alanine aminotransferase (ALT; EC 2.6.1.2) and aspartate aminotransferase (AST; EC 2.6.1.1) activities were assayed by the method of IFCC (International Federation of Clinical Chemistry). All of biochemical analyses were performed using commercial kit (Parsazmun Company, Iran) and an automated analyzer (Technicon Company, RA1000 model, Made in U.S.A).

*Pancreas weight changes*

At sacrifice, the weight of the pancreas was determined, and to reduce the individual body weight differences, the relative weight (%) was calculated using body weight at sacrifice and absolute weight as follows: [Absolute pancreas weight/Body weight at sacrifice] ×100. 

*Histopathological study*

On the last day of the experiment, pancreas and liver were removed and kept in 10% formaldehyde. Dehydration and clearing of the tissues were performed automatically using tissue processor (LEICA Company, TP 1010 model, Made in Germany). After paraffin embedding, 3-5 μm thickness serial sections were prepared using microtome (LEITZ Company, 1512 model, Made in Germany) and stained with hematoxylin and eosin (H&E) for light microscopic examination. Stained sections were quantitatively (morphometry) and qualitatively (morphology) evaluated. For quantitative analysis of pancreas, following factors were evaluated:

I: The average of pancreatic islet numbers was calculated in 10 parts of pancreatic parenchyma in each section (equivalent to 10 fields of 10x objective lens of light microscope) and 20 fields in each group. 

II: Mean diameter of the pancreatic islet was measured in 6 islets in each section and in total 12 islets in each group using an ocular micrometer and calculated with the following formula (21):

Mean diameter = √ l × b × magnification, where l is the length and b the breadth of the islets.

Approximately 10 microscopic fields were selected randomly in each liver section. Three zones can be distinguished in the portal acinus; zones 1 (Z1; periportal), 2 (Z2; central) and 3 (Z3; perivenous) from roughly concentric layers. In each selected field of vision, the histological zones were determined and morphological changes of the field were investigated. The following morphological variables were studied: changes of central vein, hepatocytes, liver plates, hepatic sinusoids, perisinusoidal space of Disse, and portal triad (hepatic artery, portal vein, and bile duct).

*Statistical analysis*

All values used in the analyses represent the mean ± SEM. Statistical analyses were performed using one-way ANOVA followed by Tukey’s multiple comparison post hoc tests. Differences were considered to be statistically significant when p was *< *0.05. All statistical analyses were performed with statistically available software (SPSS 15 for WINDOWS).

## Results

*Changes in body weight and food and water intakes*

Induction of diabetes with streptozotocin was associated with the characteristic development of a decrease in body weight and a greater food and water intakes (Table 1). In spite of increased food and water intakes, the body weights of D group were significantly decreased (p *< *0.05) with respect to N and N+G groups (Table 1). In diabetic treated groups especially in D+G_b_ group, body weight were significantly increased (p < 0.05) compared to D group (Table 1). Also, body weight of D+G_b_ group were significantly increased (p < 0.05) with respect to D+G_a_ group. In D group, a significant increase (p < 0.001) in food & water intakes was detected compared to other group, however, these abnormal increases were significantly decreased (p < 0.001) in diabetic treated groups (D+G_b_ and D+G_a_) compared to the D group (Table 1). In addition, food & water intakes of D+G_b_ group were significantly decreased (p < 0.001) with respect to D+G_a _group. 

**Table 1:** Body weight, food intake, and water intake in experimental groups

**Parameters**	**Experimental groups**					
	**N**	**N+G**	**D**	**D+Gb**	**D+Ga**	
Body weight (g)	250.37±4.48	255.50±2.79	153.50±4.80^*^	224.25±2.86^†^		198.50±3.73^§^
Food intake (g/24 h)	15.02±0.79	15.42±0.96	31.80±0.66^***^	19.62±0.78^†††^		25.20±1.45^§§§^
Water intake (ml/24 h)	20.21±1.57	26.47±1.56	132.75±7.23^***^	65.65±7.22^†††^		96.16±1.20^§§§^

Values are the mean ± S.E. for eight rats per group.

^*^ Significant difference (p<0.05) & ^***^ Significant difference (p<0.001) with N, N+G, D+G_b_ and D+G_a_ groups.

^†^ Significant difference (p<0.05) & ^†††^ Significant difference (p<0.001) with N, N+G and D+G_a_ groups.

^§^ Significant difference (p<0.05) & ^§§§ ^Significant difference (p<0.001) with N and N+G groups.

N=Normal, N+G=Normal+Garlic, D=Diabetic, D+G_b_=Diabetic+Garlic_before, D+G_a_=Diabetic+Garlic_after

*Changes in serum glucose levels*

**Figure 1:**
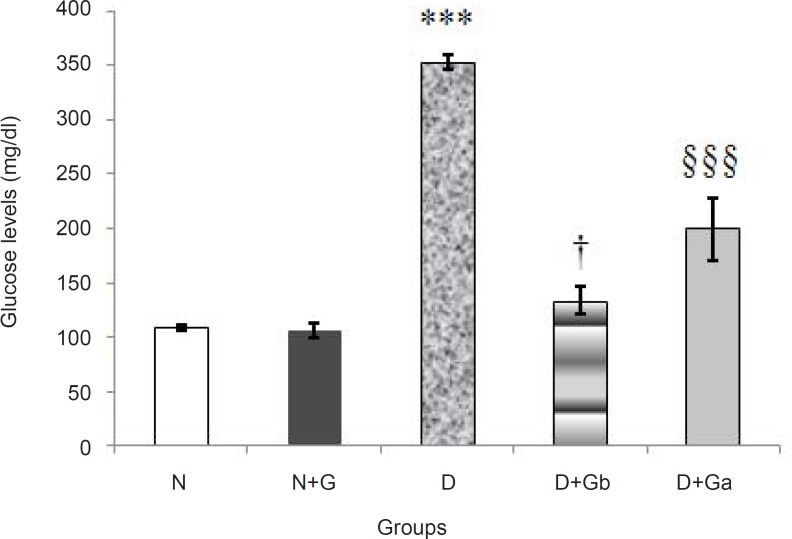
Effect of oral administration of garlic juice on serum glucose concentration. Each column represents mean ± S.E.M. for eight rats. Normal group administrated with distilled water as a vehicle. ^***^Significant difference (p<0.001) with N, N+G, D+Gb and D+Ga groups, ^†^Significant difference (p<0.05) with D+Ga group, and §§§ Significant difference (p < 0.001) with N and N+G groups. (N=Normal, N+G=Normal+Garlic, D=Diabetic, D+Gb=Diabetic+Garlic-before, D+Ga=Diabetic+Garlic-after).

In D group, a significant increase (p < 0.001) in serum glucose levels was detected compared to N and N+G groups. In comparison with D group, D+G_a _group had a significant decrease (p < 0.001), but compared to N and N+G groups (p < 0.001*) *and D+G_b _group (p < 0.05*)*, it showed a significant increase (Figure 1). In comparison with D group, D+G_b_ group had a significant decrease (p < 0.001) and there was no significant difference between D+G_b_ group and N and N+G groups. 

*Changes in serum cholesterol and triglycerides levels*

**Figure 2:**
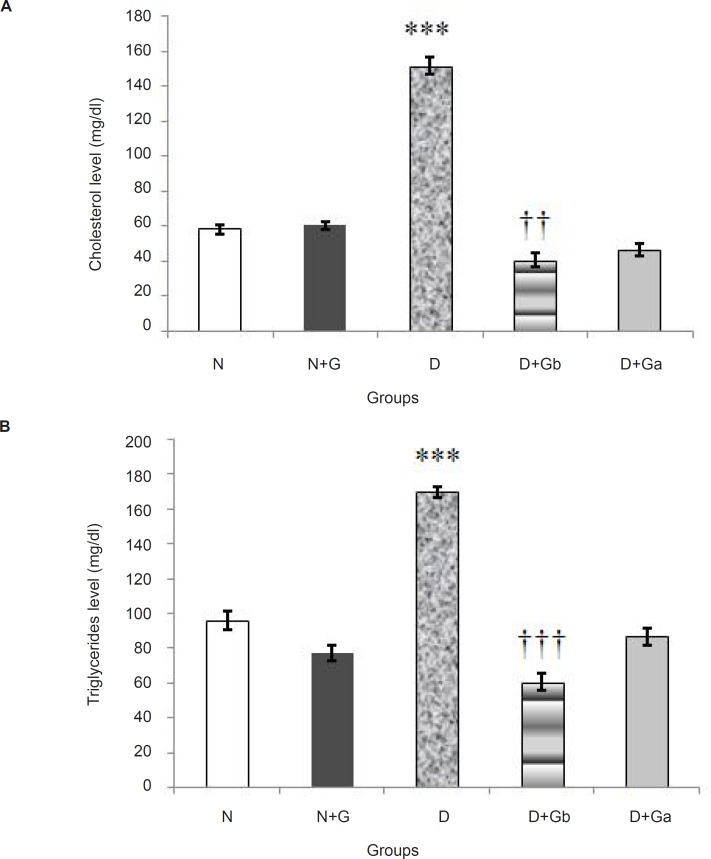
Effect of oral administration of garlic juice on serum cholesterol (A), and triglycerides (B) concentrations. Each column represents mean ± S.E.M. for eight rats. Normal group administrated with distilled water as a vehicle. ^***^Significant difference (p < 0.001) with N, N+G, D+G_b _and D+G_a_ groups in both charts. ^††^significant difference (p < 0.01) with N and N+G groups in chart A. ^†††^significant difference (p < 0.001) with N and D+G_a_ groups in chart B. (N= Normal, N+G=Normal+Garlic, D=Diabetic, D+G_b_=Diabetic+Garlic-before, D+G_a_=Diabetic+Garlic-after)

The concentrations of serum cholesterol and triglycerides are presented in Figure 2. In D group significant increases (p < 0.001) in cholesterol and triglycerides concentrations were detected compared to N and N+G groups. In D+G_a_ and D+G_b_ groups serum levels of cholesterol and triglycerides were significantly decreased with respect to D group (Figure 2A and Figure 2B). In D+G_b_ group concentration of cholesterol in comparison with N and N+G groups and concentration of triglycerides in comparison with N and D+G_a_ groups showed significant decreases respectively *(*p < 0.01*) *and (p < 0.001) (Figure 2A and Figure 2B). 

*Changes in serum alanine aminotransferase (ALT) and aspartate aminotransferase (AST) activities*

**Figure 3:**
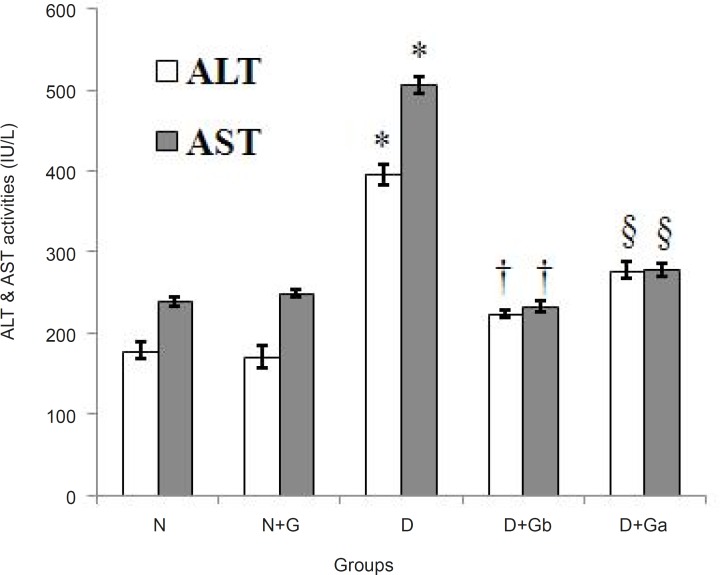
Effect of oral administration of garlic juice on the serum activities of ALT and AST. Each column represents mean ± S.E.M. for eight rats. Normal group administrated with water as a vehicle. *Significant difference (p<0.05) with N, N+G, D+Gb and D+Ga groups in both charts. †Significant difference (p<0.05) with N, N+G and D+Ga groups and §significant difference (p<0.05) with N and N+G groups in chart of ALT. †Significant difference (p < 0.05) with D+Ga group and §significant difference (p < 0.05) with N group in chart of AST. (N=Normal, N+G=Normal+Garlic, D=Diabetic, D+Gb=Diabetic+Garlic-before, D+Ga=Diabetic+Garlic-after).

The effect of garlic juice on serum ALT and AST activities are presented in Figure 3. In D group the activities of serum ALT and AST were significantly increased (p < 0.05) compared to N and N+G groups. In D+G_b_ and D+G_a_ groups the activity of serum ALT was significantly decreased (p < 0.05) compared to D group, whereas in comparison with N and N+G groups it showed a significant increase (p < 0.05) (Figure 3). Also, in D+G_b_ group, the activity of serum ALT was significantly decreased (p < 0.05) compared to D+Ga group. 

In D+G_b_ and D+G_a_ groups the activity of serum AST was significantly decreased (p < 0.05) compared to D group. The activity of serum AST in D+G_b_ group had no significant difference with N and N+G groups but in comparison with D+G_a _group it showed a significant decrease (p < 0.05). In addition, D+G_a_ group showed a significant increase (p < 0.05) compared to N group (Figure 3). 

*Changes in pancreas weight*

In diabetic rats, pancreas weight was significantly decreased (p < 0.001) compared to N, N+G, D+G_b_ and D+G_a_ groups. Although we have seen a decrease in pancreas weight, but no significant difference was seen between the D+G_b_ and D+G_a_ groups with the N group (Table 2). However, D+G_a_ had a significant difference (p < 0.01) with N+G group. To reduce the individual body weight differences, the relative weight (%) is presented in Table 2. The present study showed that the relative weight (%) were significantly decreased (p < 0.001) in diabetic rats as compared to N, N+G, D+G_b_ and D+G_a_ groups and also no significant difference was seen between other groups (Table 2). 

**Table 2:** Changes in pancreas weights in all experimental groups

**Groups **	**Pancreas weight **	
	**Absolute weights (g) **	**Relative weights **
N	0.31±0.010	0.13±0.005
N+G	0.32±0.009	0.12±0.004
D	0.13±0.018^*** ^	0.08±0.013^*** ^
D+G_b _	0.28±0.006	0.12±0.003
D+G_a_	0.27±0.003^††^	0.14±0.002

Values are the mean ± S.E. for eight rats per group.

^***^Significant difference (p*<0.001*) with N, N+G, D+G_b_ and D+G_a_ groups.

^††^ Significant difference (p*<0.01*) with N+G group. N=Normal, N+G=Normal+Garlic, D=Diabetic, D+G_b_=Diabetic+Garlic_before, D+Ga=Diabetic+Garlic_after.

*Histomorphologic changes of pancreas*

**Figure 4:**
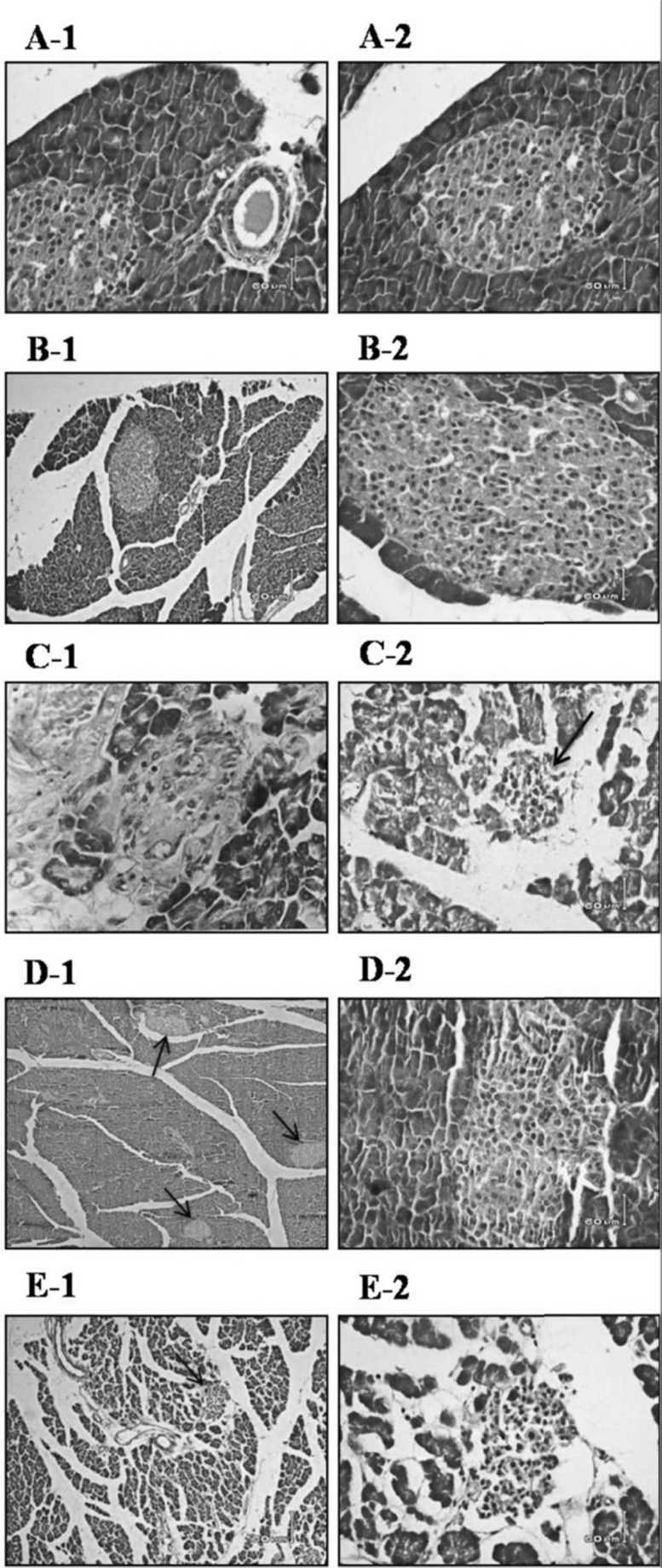
Effect of garlic juice on the structure of pancreatic islets. Nuclear changes, karyolysis, disappearing of nucleus and in some places residue of dead cells and severe necrotic changes of pancreatic islets, especially in the center of the islets were visible in D group. Islets of Langerhans in N group (A-1 & A-2), N+G group (B-1 & B-2), D group (C-1 & C-2), D+Gb group (D-1 & D-2), and D+Ga group (E-1 & E-2). The magnification of parts of A-1, A-2, B-2, C-1, C-2, D-2 & E-2 are ×40 and parts of B-1, D-1, & E-1 are ×10. Scale bars=60 μm, H&E staining. (N=Normal, N+G=Normal+Garlic, D=Diabetic, D+Gb=Diabetic+Garlic-before, D+Ga=Diabetic+Garlic-after).

In D group, decrease of pancreatic islet numbers and their mean diameter, atrophy and vacuolation and subtle invasion of connective tissues in parenchyma of pancreatic islets were detected. Pancreatic sections stained with H&E showed that diabetes caused severe necrotic changes of pancreatic islets, especially in the center of the islets. Nuclear changes, karyolysis, disappearance of nucleus and in some places residue of dead cells were visible (Figure 4, C-1). In addition, severe atrophy in the acinar parts of pancreas was detected (Figure 4, C-2). These abnormal histological signs were dramatically decreased in D+G_b_ group compared to D group (Figure 4, D-1&D-2). In D+G_a_ group compared to D+G_b_ group, slighter effects of garlic juice on the histopathological changes of the pancreas were observed (Figure 4, E-1& E-2). In N+G group in comparison with N group, an increase in pancreatic islet diameters were detected (Figure 4, B-2). 

*Histomorphometric study of pancreas*

**Figure 5:**
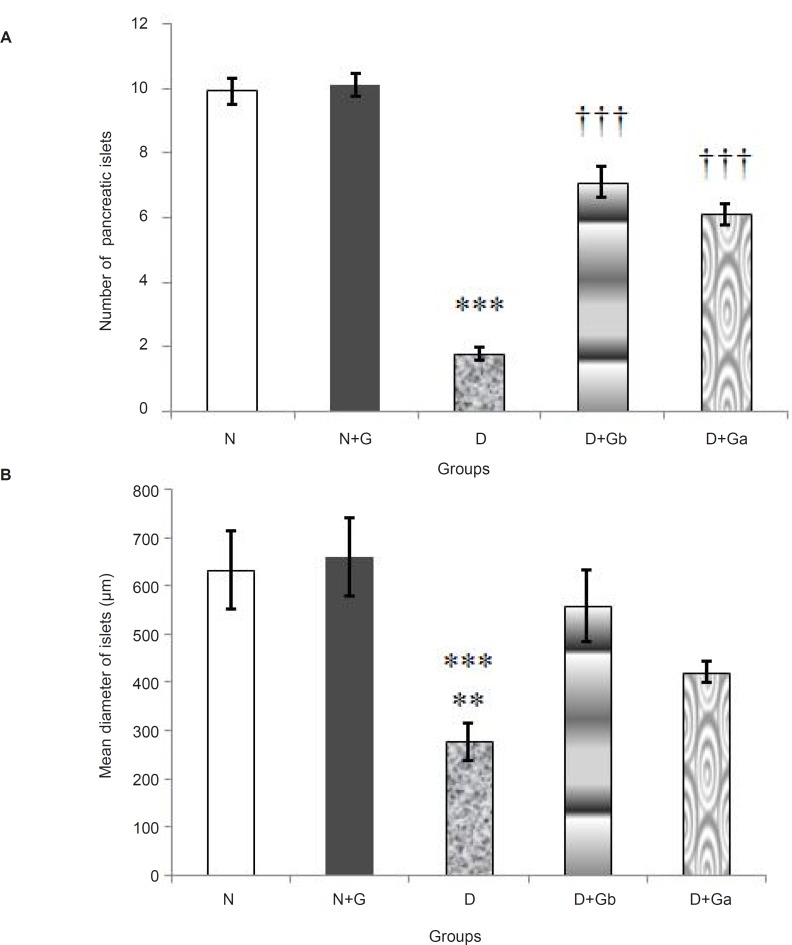
Effect of oral administration of garlic juice on number of pancreatic islets (A) and mean diameter of pancreatic islets (B). Each column represents mean ± S.E.M. for eight rats. Normal group administrated with water as a vehicle. ^***^Significant difference (p < 0.001) with N, N+G, D+G_b_ and D+G_a _groups and ^†††^Significant difference (p < 0.001) with N and N+G groups in chart A. ^***^Significant difference (p < 0.001) with N and N+G groups and ^**^Significant difference (p < 0.01) with D+G_b_ group in chart B. (N=Normal, N+G=Normal+Garlic, D=Diabetic, D+G_b_=Diabetic+Garlic-before, D+G_a_=Diabetic+Garlic-after

At histomorphometrical analysis, a significant decrease (p < 0.001) of pancreatic islet numbers were detected in D group compared to that of N, N+G, D+G_b_, and D+G_a_ groups (Figure 5A). Although, the number of pancreatic islets were significantly increased (p *< *0.001) in D+G_b_ and D+G_a_ groups compared to that of D group, they still had a significant difference (p < 0.001) with N and N+G groups. However, the number of pancreatic islets in D+G_b_ group in comparison with D+G_a_ group showed much increase (Figure 5A). 

The pancreatic islets mean diameter were significantly decreased in D group compared to that of N and N+G groups (p < 0.001) and D+G_b_ group (p < 0.01) (Figure 5B). Although, the diameter showed an increase in D+G_a_ group in comparison with D group, however, it wasn’t significant (Figure 5B). Also, there was no significant difference between D+G_b_ group and N and N+G groups. 

*Histomorphological changes of liver*

**Figure 6:**
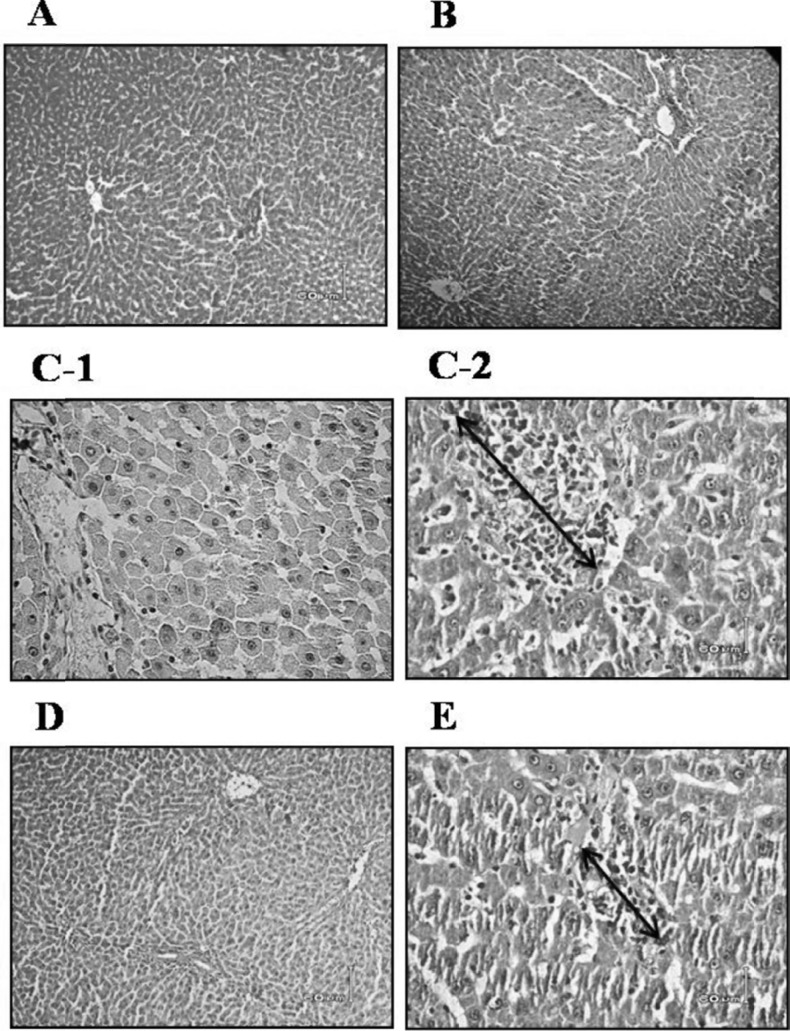
Effect of oral administration of garlic juice on the histophatological changes of the liver. Mild clearing of cytoplasm and nucleus of hepatocytes and lymphocyte infiltration in the portal areas in D group. Arrowheads indicate lymphocyte infiltration. N group (A), N+G group (B), D group (C-1 &C-2), D+G_b _group (D), and D+G_a_ group (E). The magnification of parts of C-1, C-2, & E are ×40 and parts of A, B & D are ×10. Scale bars=60 μm, H&E staining. (N=Normal, N+G=Normal+Garlic, D=Diabetic, D+G_b_=Diabetic+Garlic-before, D+G_a_=Diabetic+Garlic-after).

In D group, mild clearing of cytoplasm and nucleus of hepatocytes and their separated necrosis were observed. In addition, lymphocytic infiltration in the portal areas was seen (Figure 6, C-1&C-2). 

**Figure 7:**
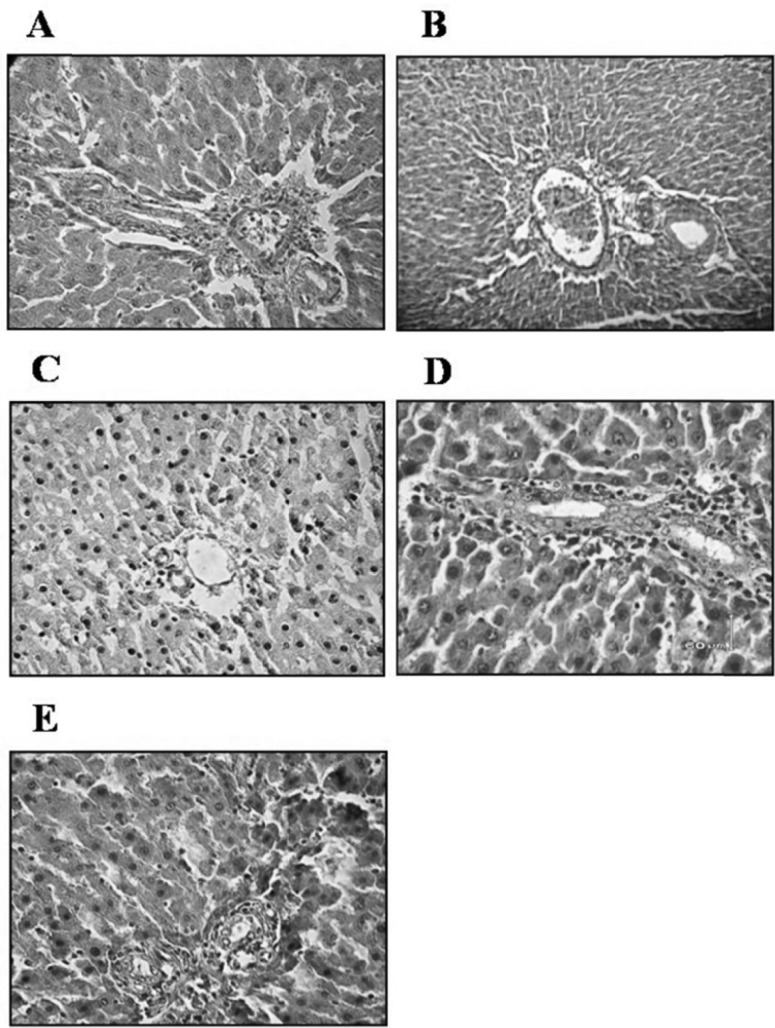
Effect of oral administration of garlic juice on the histophatological changes of the liver plates and the portal triad size. In D group, focal liver cells disruption was obvious and portal triad, to some extent, showed a decrease in volume and size. N group (A), N+G group (B), D group (C), D+G_b_ group (D), and D+G_a_ group (E). The original magnification: ×40, Scale bars=60 μm, H&E staining. (N=Normal, N+G=Normal+Garlic, D=Diabetic, D+G_b_=Diabetic+Garlic-before, D+G_a_=Diabetic+Garlic-after

Focal liver cells disruption was obvious (Figure 7C). In D group sinusoids showed little changes and there was also apoptosis of 2-3 hepatocytes per 10 high power field (HPF) of light microscope. Portal triad, to some extent, showed a decrease in volume and size (Figure 7C). In our study, in D group, inclusions in the nuclei of hepatocytes and steatosis into the hepatocytes weren’t seen. In D+G_b_ and D+G_a_ groups in comparison with D group, all of the pervious signs had a better improvement. However, in D+G_a_ group, we observed lymphocytic infiltration and dispersal of liver plates (Figure 6E). In general, D+G_b_ group was in a better condition than D+G_a_ group, and it had almost returned to the normal levels. N+G group showed subtle increase in size of portal triad in comparison to the N group however, it was not significant (Figure 7B).

## Discussion

Aside from its general use as a condiment, garlic (*Allium sativum*) is known for its pharmacological and nutritional properties. Garlic has long been believed to possess a hypoglycemic effect (11, 12); however, both effectiveness and ineffectiveness of garlic preparations on decreasing blood glucose have been reported (22-25). In the present study, we investigated the preventive and therapeutic effect of garlic juice on diabetes mellitus in a widely used animal model of diabetes.

In diabetic rats, elevated levels of glucose, cholesterol and triglycerides (TG), the increment of the activities of ALT and AST, increased food & water consumption and decreased body weight were observed. Interestingly, garlic juice administration significantly normalized the blood glucose levels to that of control and ameliorated increased serum levels of cholesterol and triglycerides and increased serum activities ALT and AST. In comparison with diabetic rats, there was significant in the states of polyphagia, polydipsia and body weight with garlic treatment. 

The possibility of glucose-lowering effects of garlic was supported experimentally in a number of studies. The first report considering the beneficial effects of allicin, a biologically active sulfoxide from garlic in diabetic mice was published as far back as in 1973 (26). Further studies have demonstrated that sulfur-containing amino acids from garlic possess a direct hypoglycemic action, potentiate the effects of insulin on the body, and increase the hepatic glycogen synthesis in diabetic mice and rabbits (13, 14, 27). 

Since a direct stimulatory effect of S-allyl cysteine sulfoxide (SAC), a garlic compound, which is a precursor of garlic oil on insulin secretion of pancreas has been shown (15, 28), it is also possible that garlic juice stimulates insulin secretion directly as ameliorative effects of garlic juice on fasting blood glucose was found in the present study. 

Another proposed mechanism is to spare insulin from sulfhydryl group. Inactivation of insulin by sulfhydryl group is a common phenomenon. Garlic can effectively combine with compounds like cysteine and enhance serum insulin (26). 

Atherosclerosis, by far the greatest killer in modern society, is a complex disease that develops because of many risk factors including alterations in plasma lipids and lipoprotein levels, blood pressure regulation, platelet function, clotting factors, arterial smooth muscle cell metabolism, and *etc*. (29). Among all risk factors of atherosclerosis, diabetes mellitus is thought to be one of the most potent that greatly increases the risk of cardiovascular diseases of atherosclerotic origin (30).

Historically, there has been great interest in the role of garlic in reducing cardiovascular risk factors, but there was little scientific support of its therapeutic and pharmacologic properties until recently, when the effects of garlic have been extensively evaluated (31, 32).

In diabetic rats increased triglycerides and cholesterol levels were observed. Moreover, the lipid content of cell membranes seems to be disrupted by diabetes as proved by increased non-enzymatic glycation, lipid peroxidation and cholesterol/phospholipid ratio (33). 

Raised nonesterified fatty acid concentrations in plasma have been proposed as a major cause of insulin resistance and may reduce the secretion of insulin in diabetes (34). Therefore, the improved *in vivo *hypoglycemic response to the lack of insulin in the garlic juice-treated diabetic rats may be partly explained by lowered lipid concentrations in blood. Allicin accounts for approximately 70% of total thiosulfinates produced (35), when the pro-drugs alliin, isoalliin and methiin interact with allinase after mature garlic bulbs are crushed or garlic powder is mixed in water (36). Dietary supplements unable to produce allicin are associated with a lack of blood cholesterol-lowering efficacy (37). Thus maximization of allicin yield seems a key component in relation to lowering elevated blood cholesterol levels. Our study utilized garlic juice, which is a rich source in terms of allicin releasing potential.

Garlic may also decrease complications of diabetes such as cardiovascular disease risk if it lowers plasma TG levels. In fact, some clinical studies have shown that garlic or garlic preparations did lower plasma TG levels (38, 39). Previous studies indicated that a TG-lowering effect of garlic extracts might stem in part from inhibition on hepatic TG synthesis (32, 40).

Although garlic and garlic preparations have been shown to reduce (38, 41, 42) or have no effect (43, 44) on plasma concentration of cholesterol and TG, in this study, we reported a reduction in serum levels of cholesterol and triglycerides, which was less than normal level in rats treated with garlic juice for 3 weeks before STZ injection (D+G_b_ group). Whether the high magnitude of the reduction in plasma cholesterol concentration is attributable to high tissue level of potential active components of garlic remains to be established. The reason for the contradictory observations of the effect of garlic on cholesterol is uncertain, but it may be explained in part by differing experimental designs. These conflicting data may, at least in part, be due to differences in the way the garlic was prepared, the route of administration, the dose given, and the duration of treatment. Hence, it is difficult to determine whether garlic truly modulates plasma lipids under conditions relevant to human.

Most research on liver in diabetes mellitus has focused on the biochemical and pathophysiological changes; however in comparison, histological aspects of liver structure have received less attention. Also, reports on pathology have focused mainly on late changes in diabetes, viz., glycogenosis, fatty liver and cirrhosis of the micronodular variety (45, 46); early qualitative structural changes in the liver have not been studied well.

Since liver is a major site of drugs and waste products detoxification (47, 48) and also as repeated administration of streptozotocin in patients with pancreatic neoplasm induces hepatotoxicity only occasionally (49, 50), we therefore believe that the changes observed were due to the induction of diabetes and not only due to the toxic effect of streptozotocin.

Diabetes has been shown to be a state of increased free radical production. Mechanisms that contribute to the formation of free radicals in diabetes may include not only increased non-enzymatic and auto-oxidative glycosylation, but also metabolic stress resulting from changes in energy metabolism, levels of inflammatory mediators, and the status of antioxidant defense (22).

Evidence is accumulating suggesting that the free radicals play a crucial role in the streptozotocin-induced diabetes. Damasceno *et al. *(51) and Gul *et al. *(52) reported that streptozotocin produced oxidative stress and depletion of antioxidant systems in both blood and tissues particularly, liver. STZ-induced hepatic toxicity is thought to be mediated by ROS which produces lipid peroxide in hepatocellular membrane (53, 54).

In our study, we also observed mild clearing of cytoplasm and nucleus of hepatocytes and lymphocytic infiltration in the portal areas (Figure 6, C-1&C-2). These abnormal histological signs were dramatically decreased in D+G_b_ group compared to D group. In D+G_a_ group compared to D+G_b_ group slighter effects of garlic juice on the histopathological changes of the liver were observed.

In addition, chronic hyperglycemia causes elevated concentrations of reactive oxygen species accompanied by lowered enzymatic and nonenzymatic cell antioxidant defenses. ROS have been suggested to be involved in pancreas *β*-cell dysfunction and insulin resistance (55).

Garlic extracts elicit antioxidant action by scavenging ROS, enhancing the cellular antioxidant enzyme superoxide dismutase, catalase and glutathione peroxidase, and increasing glutathione in the cells (56).

On the other hand, El-Demerdash *et al*. (18) showed that the increase in the activities of plasma AST, ALT, lactate dehydrogenase (LDH), alkaline phosphatase (AlP), and Acid phosphatase (AcP) may be due to the induction of hepatic dysfunction by diabetes. 

For this reason, the other aims of the present work were also to look at the effect of ingesting garlic juice on the serum activities of amino transferases (AST and ALT). Diabetic rats treated with garlic juice showed a significant reduction in the serum activities of aminotransferases (p < 0.05) as compared to the diabetic rats. 

The increased protein catabolism accompanying gluconeogenesis and urea formation that are seen in diabetic state might be responsible for the elevation of these tissue transaminases. The rise in the activity of alanine transaminase is due to hepatocellular damage and is usually accompanied by a rise in aspartate transaminase (57).

In addition, Bopanna *et al. *(58) and Eskander *et al*. (59) demonstrated that administration of several herb extracts in diabetes could restore the changes in the activities of serum enzymes like ALP, AcP and transaminases: AST and ALT. Also, in the present study, treatment of the diabetic rats with garlic juice caused reduction in the activity of ALT and AST in plasma compared to the mean values of diabetic group. These results are in agreement with those obtained by Ohaeri (60) in rats. Nakagawa *et al. *(61) have also demonstrated liver protection by S-alk(en)ylcysteins and alliin on hepatocytes *in-vitro*. Therefore, garlic can also be used for liver protection. 

Generally, body weight decreases as diabetes progresses, but garlic juice significantly inhibited the decrease in body weight from the start of administration. Inhibition of body weight loss is also considered an indication of the antidiabetic activity of garlic. 

In addition, absolute pancreas weights decreased in diabetic rats as compared to the other groups, meaningful changes were detected in relative weights. Therefore, the decrease in absolute weights detected can be considered to result from diabetic changes and not due to the decrease in body weights.

In diabetic rats, inflammatory changes detected in pancreatic islets result from selective destruction of insulin-producing *β*-cells, and consequently, the atrophy, vacuolation and the decrease in the number of pancreatic islets were observed (62). Atrophy and vacuolation of islet cells according to the destruction of *β*-cells were demonstrated in the present study by the decrease in the number and diameter of pancreatic islets observed in histomorphometry. The inhibition of morphology and histomorphometrical changes in the pancreas due to garlic juice administration are considered to be the direct evidence that garlic juice improves diabetes. 

In conclusion, the results of the present study show that the long-term treatment with garlic juice can improve glycemic control via improved pancreas function. Garlic juice can ameliorate increased serum levels of cholesterol and triglycerides and serum activities of ALT and AST. Generally, D+G_b_ group was in a better condition than D+G_a_ group, and it had almost returned to the normal levels. Therefore, we suggest that garlic juice may help in both prevention and protection against the complications of diabetes.
