# Expression of Concern for Preventive Effect of Garlic (*Allium sativum* L.) on Serum Biochemical Factors and Histopathology of Pancreas and Liver in Streptozotocin- Induced Diabetic Rats [IJ Pharm Res. 2013; 12(3): e125679]

**DOI:** 10.5812/ijpr-168505

**Published:** 2025-11-15

**Authors:** Editor-in-Chief IJPR

**Affiliations:** 1 School of Pharmacy, Shahid Beheshti University of Medical Sciences, Tehran, Iran

Dear Readers,

As Editor-in-Chief of the IJ Pharmaceutical Research, I wish to inform our readers of a matter currently under investigation concerning one of the published articles (1).

The journal has received a report from a whistleblower, alleging substantial similarities between this article and a previously published study from 2009, available at the following link:

https://ppj.phypha.ir/article-1-537-en.html

Following this notification, the editorial board, on behalf of the Editor-in-Chief, conducted an initial assessment. Our preliminary review indicates that although the texts are not identical, and the 2013 article includes new information—such as a broader analysis of serum biochemical factors (cholesterol and others) and histopathological examination of the liver in addition to the pancreas—there appear to be overlapping foundational descriptions and methodological components between the two studies. We also note that the 2009 article has not been cited in the 2013 publication, despite the apparent methodological similarities.

It is important to note that the earlier article was published in Persian, and due to the language difference, performing an exact and high-confidence similarity comparison between the two articles has been challenging for the editorial office. This has made the assessment process more complex and requires additional verification.

In accordance with the Committee on Publication Ethics (COPE) guidelines, the journal has initiated a formal investigation into this matter. This process involves a thorough examination of the concerns raised, communication with the authors, and evaluation of all relevant materials.

An update will be provided to our readers as soon as the investigation is complete and a final decision has been reached.

We appreciate the vigilance of the scientific community in helping maintain the integrity of the scholarly record.

Elham Rezaee

School of Pharmacy, Shahid Beheshti University of Medical Sciences, Tehran, Iran

Editor-in-Chief of IJ Pharmaceutical Research
